# Birth order modifies the effect of *IL13 *gene polymorphisms on serum IgE at age 10 and skin prick test at ages 4, 10 and 18: a prospective birth cohort study

**DOI:** 10.1186/1710-1492-6-6

**Published:** 2010-04-20

**Authors:** Ikechukwu U Ogbuanu, Wilfried J Karmaus, Hongmei Zhang, Tara Sabo-Attwood, Susan Ewart, Graham Roberts, Syed H Arshad

**Affiliations:** 1 Department of Epidemiology and Biostatistics, Norman J Arnold School of Public Health, University of South Carolina, USA; 2 Department of Environmental Health Sciences, Norman J Arnold School of Public Health, University of South Carolina, USA; 3 College of Veterinary Medicine, Michigan State University, East Lansing, Michigan, USA; 4 David Hide Asthma and Allergy Research Centre, St Mary's Hospital, Isle of Wight, and University of Southampton, Southampton, UK

## Abstract

**Background:**

Susceptibility to atopy originates from effects of the environment on genes. Birth order has been identified as a risk factor for atopy and evidence for some candidate genes has been accumulated; however no study has yet assessed a birth order-gene interaction.

**Objective:**

To investigate the interaction of *IL13 *polymorphisms with birth order on allergic sensitization at ages 4, 10 and 18 years.

**Methods:**

Mother-infant dyads were recruited antenatally and followed prospectively to age 18 years. Questionnaire data (at birth, age 4, 10, 18); skin prick test (SPT) at ages 4, 10, 18; total serum IgE and specific inhalant screen at age 10; and genotyping for *IL13 *were collected. Three SNPs were selected from *IL13: *rs20541 (exon 4, nonsynonymous SNP), rs1800925 (promoter region) and rs2066960 (intron 1). Analysis included multivariable log-linear regression analyses using repeated measurements to estimate prevalence ratios (PRs).

**Results:**

Of the 1456 participants, birth order information was available for 83.2% (1212/1456); SPT was performed on 67.4% at age 4, 71.2% at age 10 and 58.0% at age 18. The prevalence of atopy (sensitization to one or more food or aeroallergens) increased from 19.7% at age 4, to 26.7% at 10 and 41.1% at age 18. Repeated measurement analysis indicated interaction between rs20541 and birth order on SPT. The stratified analyses demonstrated that the effect of *IL13 *on SPT was restricted only to first-born children (p = 0.007; adjusted PR = 1.35; 95%CI = 1.09, 1.69). Similar findings were noted for firstborns regarding elevated total serum IgE at age 10 (p = 0.007; PR = 1.73; 1.16, 2.57) and specific inhalant screen (p = 0.034; PR = 1.48; 1.03, 2.13).

**Conclusions:**

This is the first study to show an interaction between birth order and *IL13 *polymorphisms on allergic sensitization. Future functional genetic research need to determine whether or not birth order is related to altered expression and methylation of the *IL13 *gene.

## Introduction

Atopy has been defined as the propensity of an individual to produce IgE in response to allergen and a predisposition to the development of allergic diseases, such as asthma, atopic dermatitis, food allergy or hay fever. It is defined operationally by elevations in serum levels of immunoglobulin E (IgE) reactive with allergens or skin test reactivity to allergens [1]. Thus atopy is strictly linked to IgE production and describes the personal or familial propensity to become sensitized and produce IgE antibodies in response to environmental triggers [2].

The documented increase in childhood asthma and other atopic disorders over the past three decades has necessitated a search for possible underlying mechanisms and mediators [3,4]. Genetic variation has been documented to play a role; however, when found, gene effects for allergic diseases are typically small and not easily replicable [1]. In addition, recent studies reveal that a change in the genetic pool is insufficient to account for the temporal and spatial correlates of this increase in prevalence, and suggest that modifications of gene expression and function may be more important [5,6]. Such modifications of gene expression are thought to result from gene-environment interactions occurring before and after birth. Indeed, previous evidence suggests that developmental processes may modify the impact of genetics on atopy development later in life [7,8]. Thus, current etiological research is focusing on modifiers of gene effects as possible precipitating factors for the increasing prevalence of atopy in childhood.

Previous studies have assessed two related ideas - the hygiene hypothesis and the birth order effect [9]. The hygiene hypothesis suggests that exposure to infections after birth (due to transmission from older siblings or other children), may influence the development of the immune system along a non-allergic (T helper 1; Th1) pathway, leading to a reduced risk of asthma and other allergic diseases. Although still under investigation, the hygiene hypothesis may explain observed associations between family size, birth order, day-care attendance, and the risk of asthma and allergy. According to the hygiene concept, the effect of birth order is through sibling hierarchy, where the younger child is prone to infection from the older sibling and hence is at lower risk of atopy.

Other studies suggest that birth order may act through a different mechanism [9]. *In utero *sensitization as a possible mechanism of modification of gene effects has been proposed, where birth order, as an indicator of variations in prenatal exposure, acts independent of the *number *of siblings [10,11]. A previous report by our group [12] suggested that the birth order effect may result from *in utero *exposure as indicated by changes in maternal IgE [12]. If a protective effect of birth order is already present at the time of birth of the child, then the effect of hygiene later in childhood may not be as relevant as is currently thought. In support of this explanation, other non-allergic diseases such as preeclampsia are more common in first born children or first pregnancies, when compared to subsequent births or pregnancies, [13] suggesting altered *intra uterine *immune adaptation as a possible underlying mechanism.

In a recent review, Vercelli [1] suggested that one of the major challenges facing geneticists is to understand how environmental and developmental factors interact with genetic determinants to increase disease susceptibility. Previous studies have assessed the individual effects of birth order [10,14,15] and *IL13 *polymorphisms [16-21] on allergic phenotypes. It is noteworthy that none of these reports examined the possible interaction between birth order and *IL13 *polymorphisms on childhood atopy.

In previous reports by our group and other researchers, birth order has been repeatedly found to be associated with atopic markers. For instance, we found that cord serum IgE was reduced in offspring with higher order, indicating that the sibling effect may have its origin *in utero *[12], before any hygiene effect in early childhood could affect the offspring. In addition, we found that the number of live offspring significantly reduces maternal IgE [22], which supports the idea that maternal immune tolerance against allergens may increase with increasing order of live offspring; this may transmit a lower risk of developing atopy to children of a higher birth order.

Our *a priori *hypothesis was that the birth order effect may interact with the genetic predisposition of the offspring in influencing future atopic manifestations. In order to test this hypothesis, we chose one of the most-studied "atopy genes," *IL13*. This choice seems appropriate because, if a gene-by-birth order interaction exists, it would be best demonstrated with a known gene. Finding such an interaction with a novel gene that does not have the kind of consistent replication so far demonstrated for *IL13 *may not be as scientifically robust.

This report will be the first study to investigate the interaction between these important risk factors during early childhood, as well as sustained effects in adolescence, testing both the main effects and the interaction effects of the *IL13 *gene with prenatal factors.

## Materials and methods

### Study population

The study participants were mother-infant pairs enrolled in the 1989 Isle of Wight birth cohort study. This study represents an unselected whole population birth cohort based on the Isle of Wight, U.K. The Isle of Wight is an island (13 × 23 miles) just off the South Coast of England with a resident population of 130,000. The population is stable to the extent that the majority of participants in the cohort has not moved away and was thus available for follow up. This makes the island particularly attractive for long-term prospective epidemiological studies.

Between January 1989 and February 1990, children born on the Isle of Wight, U.K. were recruited to participate in a longitudinal study (n = 1,456). The local Research Ethics Committee approved the study and informed written parental consent was obtained for all the participants at recruitment and subsequently at each follow up. This whole-population birth cohort was largely Caucasian (99%) and was living in a semi-rural, non-farming environment with no heavy industry.

The Isle of Wight birth cohort has been described in detail elsewhere [23,24]. Briefly, pregnant women were recruited prenatally and data from birth records and extensive questionnaires were collected, including information on family history of asthma and allergy, as well as maternal smoking habits. At ages 1, 2, 4, 10 and 18 years, the original questionnaire-based information was updated, a study investigator performed physical examinations on the children, and symptoms of asthma and allergic diseases were recorded. Skin prick tests (SPT) to common food and aero-allergens were administered at the 4, 10 and 18 year follow-up visits [25], and at age 10, anti-coagulated blood samples were collected and stored frozen for subsequent DNA analysis. Also at age 10, total serum IgE and inhalant IgE screen were performed in the blood samples of the children. Further information was collected at the 10 and 18 year visits using standardized International Study of Asthma and Allergy in Childhood (ISAAC) questionnaires.

## Study variables

This is a population-based association study that tests the interaction effects between *IL13 *SNPs and birth order on atopic outcomes. Thus, the main exposure variables involved in the interaction terms were *IL13 *polymorphisms and birth order. Outcome variables include skin prick tests at ages 4, 10, and 18, total IgE at birth (cord serum) and at age 10, and inhalant IgE screen at age 10. The potential confounders we adjusted for were gender, environmental tobacco smoke exposure, family SES cluster, and gestational age. Gender was classified into male and female while gestational age was assessed in weeks. Other variables are described in greater detail below.

### *IL13 *genotyping

Genomic DNA was isolated from blood samples using QIAamp DNA Blood Kits (Qiagen, Valencia, CA) or the ABI PRISM™ 6100 Nucleic Acid PrepStation (Applied Biosystems, Foster City, CA). Polymorphisms in the *IL13 *gene were examined using the SNPper and Applied Biosystems databases. Genotyping was conducted by fluorogenic 5' nuclease chemistry PCR using Assays on Demand kits cycled on a 7900HT Sequence Detection System (Applied Biosystems, Foster City, CA), or biotin-streptavidin-based pyrosequencing performed on PSQ-96 instrumentation (Biotage AB, Uppsala, Sweden).

*IL13 *is a small gene (2.9 kb) characterized by extensive linkage disequilibrium, thus genotyping a few polymorphisms provided adequate assessment of genetic associations. Five *IL13 *SNPs were genotyped (Table 1): rs1800925 at the promoter region, rs2066960 at intron 1 and rs1295686 at intron 3 positions, rs20541 at the coding exon 4 position ([G] Arg to [A] Gln at amino acid position 144) and rs1295685 at the 3' UTR/exon 4 position of the gene. Each of these SNPs had a minor allele frequency ≥ 19%.

Using the Haploview program [26], three of the five single nucleotide polymorphisms (SNPs) of the interleukin 13 gene were in a linkage disequilibrium (LD) block. SNP selection was carried out using the tag-SNP approach. Since rs1295686, rs20541 and rs1295685 were in the same LD block (Figure 1 in [27]), we selected rs20541 for further analysis. In addition to being the non-synonymous SNP among these three, it is also the *IL13 *SNP that has been most studied [17,21,28-31].

**Table 1:** Genotype proportions for *IL13 *single nucleotide polymorphisms (SNPs)

SNPs	Position (bp)	Location	Genotype	Frequency (%)
rs1800925	132,020,708	Promoter	CC	577 (63.6)
(-1112C/T)			CT	295 (32.5)
			TT	35 (3.9)
			Total	907 (100.0)
				
rs2066960	132,022,334	Intron 1	CC	729 (81.5)
			AC	157 (17.6)
			AA	8 (0.9)
			Total	894 (100.0)
				
rs1295686	132,023,742	Intron 3	CC	483 (64.6)
			CT	240 (32.1)
			TT	25 (3.3)
			Total	748 (100.0)
				
rs20541	132,023,863	Exon 4	GG	583 (64.4)
(R130Q)			GA	291 (32.1)
			AA	32 (3.5)
			Total	906 (100.0)
				
rs1295685	132,024,344	Exon 4	GG	584 (64.5)
			GA	280 (30.9)
			AA	41 (4.5)
			Total	905 (100.0)

### Birth order

Birth order was obtained from the questionnaire data collected at birth. We defined three groups for this analysis: "first", "second", and "third and higher" order birth positions. Birth order equals the number of older siblings plus one [12].

### Skin Prick Testing (SPT)

Skin prick testing to 14 common food and aero-allergens was performed at 4 (n = 981), 10 (n = 1036) and 18 years (n = 845) using a standard battery of food and aeroallergens (ALK, Horsholm, Denmark), which have been previously described [32]. After 15 minutes, a mean wheal diameter ("sum of longest diameter and diameter diagonal to it" divided by 2) of at least 3 mm greater than the negative control was considered evidence of sensitization. Sensitization to at least one food or aeroallergen was recorded as a positive reaction to SPT.

### Serum Total IgE Determination

Total IgE was measured in samples of cord serum (n = 1340) and serum collected at age 10 (n = 923). Total IgE in cord serum was measured using Pharmacia IgE EIA^® ^(Pharmacia Diagnostics AB, Uppsala, Sweden) [33], which is designed to measure IgE between 0.2 to 50 kU/L on 0.1 ml of serum or plasma [34,35]. Maternal IgE and IgE at age 10 were determined using PRIST^® ^(Pharmacia Diagnostics AB, Uppsala, Sweden) designed to measure IgE between 2.0 to 1000 kU/L. For our analysis, maternal IgE was dichotomized into < 100 and ≥ 100 kU/L.

### Maternal Atopic Status

Using information from questionnaires on maternal atopic history (Yes vs. No; n = 1213) and data from the measured maternal IgE level at birth (n = 1037), we created a composite variable with four levels: "definite" maternal atopic status (elevated IgE, positive history of atopy, n = 163); "latent" (elevated IgE, negative history, n = 201); "probable" (normal IgE, positive history, n = 121); and "none" (normal IgE, negative history, n = 552).

### Serum Specific Inhalant IgE Screening

The inhalant screen was a non-quantitative test for specific allergens. The test was positive if it detected antibodies against one or more of the following allergens; house dust mite (*D. pteronyssinus *and *D. farinae*), cat dander, dog dander, horse dander, timothy grass, cladosporium, silver birch, olive, mugwort and nettle. Blood samples were allowed to stand and coagulate in Gel and Clot Activator tubes (Vacutainer Systems, Europe) for at least 10 minutes. They were then centrifuged at 3000 revolutions per minute for a further 15 minutes. Serum was then stored at minus 40°C until analysis for serum IgE. Results were recorded as either positive or negative to inhalant IgE screen. Individuals were classified as positive if they had IgE to one or more of the above tested aero-allergens.

### Family Social Status Cluster

"Family social status cluster" is a composite variable that accounts for "socio-economic status" broadly defined [36]. The Isle of Wight population has been characterized as semi-rural, with most families (63%) residing in "owner-occupied" homes that have been owned by their families for decades. In order to correctly classify "social status," we chose to cluster family social status using the following three variables: a) the British socioeconomic classes (1 - 6) derived from parental occupation reported at birth; b) the number of children in the index child's bedroom (collected at age 4); and c) family income at age 10. This composite variable captures the family social class across the entire study period and has been described in more detail elsewhere [36].

### Environmental Tobacco Smoke Exposure

Information on tobacco smoking by mothers (during pregnancy and later), by fathers or any other individual inside the home was recorded at recruitment and updated at each follow-up. Exposures to environmental tobacco smoke (ETS) in the household and maternal smoking during pregnancy were combined and classified into three groups. When mothers did not smoke during pregnancy and there was no exposure to household ETS in children up to the age of 10 years, children were categorized as "ETS-0". When mothers did not smoke during pregnancy but household members smoked within the home at some point up to the child's age of 10 years, the exposure status was categorized as "ETS-1". When mothers smoked during pregnancy and the children were also exposed to household ETS at some point up to the age of 10 years, the exposure was categorized as "ETS-2". None of the children had mothers who smoked during pregnancy with no exposure to household tobacco smoke after birth [28].

## Data analysis and statistics

Serum total IgE at age 10 (dichotomized into ≤ 200 kU/L and >200 kU/L) and inhalant IgE at age 10 (dichotomized into positive or negative) were analyzed as binary outcomes. Because SPT was measured at ages 4, 10 and 18 years, the three measurements for each individual child were correlated over time. To account for these correlations, we estimated the effect of birth order, *IL13 *polymorphisms, and their interaction on SPT, using generalized estimating equations (GEE). In addition, since SPTs are prevalent outcomes, we did not estimate odds ratios but prevalence ratios (PROC GENMOD with the REPEATED statement and LOG link function in SAS). The association of birth order with IgE and the inhalant screen measured at age 10 was also determined using prevalence ratios (PRs).

In order to exclude the possible confounding effect of correlated variables, multivariable regression was used to explore the predictive effect of each variable, adjusting for all potential confounders. Assessment of interaction was carried out using backward elimination from the full model (which contained three birth order by *IL13 *SNP interaction terms). The significance level for the interaction effects was set at an alpha level of 0.1, for interaction on a multiplicative scale (log-linear models), and results were presented by stratification when the "birth order by gene" effect was significant. Stratification assesses interaction between *IL13 *and birth order on an additive scale, meaning that the combined effect is more than the *sum *of the single effects, whereas multiplicative interaction implies that the combined effect is more than the *product *of the individual effects.

Following the pattern of previous publications [27,37,38], we estimated the effect of *IL13 *polymorphisms using the dominant model, i.e. the heterozygous genotype was combined with the homozygous minor allele genotype in one category, while the homozygous common allele served as the referent group. This classification was also necessary because relatively few individuals were homozygous for the risk/minor allele, as has been reported and proposed in previous reports of the *IL13 *gene [38].

## Results

### Participant demographic characteristics

Of the available 1536 children born during the recruiting period, 94.8% (1456/1536) consented to participate in this study. Birth order information was available for 83.2% (1212/1456) of the original birth cohort; skin prick test was performed on 67.4% (981/1456) of the original cohort at age 4, 71.2% (1036/1456) at age 10 and 58.0% (845/1456) at age 18 years. Of all the children who were skin-prick tested, the prevalence of atopy (positive reaction to one or more allergens) was 19.7%, 26.7% and 41.1% at ages 4, 10 and 18 years respectively.

Serum total IgE measurements at age 10 were available for 65.5% (953/1456) of the original cohort. Comparison of the demographic data between children with and without IgE measurements at age 10 revealed no evidence of selection bias in the major variables used for this analysis; however, children with no IgE measurements had greater environmental tobacco smoke exposure and were more likely to come from the low family social status cluster (Table 2).

**Table 2:** Comparison between children with and without total serum IgE measurements at age 10

	IgE at age 10 (n (%))	No IgE at age 10 (n (%))	Exact
Study Variables	N = 953	N = 583	p-values
Birth order			
- First	354 (41.0)	156 (44.7)	0.50
- Second	303 (35.1)	116 (33.2)	
- Third or higher	206 (23.9)	77 (22.1)	
Family Social Status Cluster*			
- High	80 (8.5)	31 (7.5)	**0.02**
- Middle	737 (78.0)	300 (72.8)	
- Low	128 (13.5)	81 (19.7)	
Environmental Tobacco Exposure**			
- ETS-0	451 (47.5)	196 (36.0)	**<0.0001**
- ETS-1	300 (31.6)	164 (30.1)	
- ETS-2	199 (21.0)	185 (33.9)	
Gender			
- Male	479 (50.3)	306 (52.7)	0.37
- Female	474 (49.7)	275 (47.3)	
Prematurity (weeks)			
- < 37	26 (2.8)	23 (4.1)	0.23
- ≥ 37	904 (97.2)	544 (95.9)	
Low Birth Weight (g)			
- < 2500	33 (3.6)	28 (4.9)	0.23
- ≥ 2500	894 (96.4)	539 (95.1)	

*"Family social status cluster" is a composite variable derived from a combination of family income, parental occupation (socioeconomic status), and number of children in child's bedroom.

** ETS-0: mother did not smoke during pregnancy and children not exposed to household environmental tobacco smoke (ETS);

ETS-1: mother did not smoke during pregnancy, but children were exposed to household ETS;

ETS-2: mother smoked during pregnancy and children were exposed to household ETS.

### Association of birth order and *IL13 *polymorphisms with total serum IgE and inhalant IgE screen at age 10, and with skin prick test positivity at ages 4, 10 and 18 years

#### Univariable analysis

Univariable analysis showed no significant association between birth order and IgE (serum total IgE and serum specific inhalant IgE screen positivity) at age 10 years (Table 3). Unadjusted bivariable analysis also showed no evidence of an association between birth order and SPT at ages 4, 10 and 18 years (Table 4). In addition, *IL13 *polymorphisms were not significantly correlated with atopic markers at ages 4, 10 and 18 years, except for rs2066960, which was a significant predictor of elevated serum IgE (>200 kU/L) at age 10 years (p = 0.011) (Table 3).

Other significant predictors of elevated serum IgE at age 10 years in the univariable analysis included maternal IgE (p = 0.009), maternal atopic status (p = 0.006), elevated cord serum IgE (p = 0.004) and prematurity (p = 0.008) (Table 3). Similarly, maternal history of atopy, maternal IgE, maternal atopic status and gender were significantly associated with SPT at ages 10 and 18, but not at age 4 years (Table 4). Only elevated cord serum IgE was significantly associated with skin test positivity at 4 years.

**Table 3:** Univariate analysis of the association of serum IgE and inhalant IgE screen positivity with *IL13 *and prenatal factors

	Elevated serum IgE (age 10)		Inhalant IgE +ve (age 10)	
	n (%)	Exact	n (%)	Exact
Study Variables	N = 953	p-values	N = 952	p-values
rs1800925:				
- TT/CT	108 (33.0)	0.086	117 (35.9)	0.191
- CC	173 (27.6)		197 (31.5)	
rs2066960:				
- AA/AC	62 (38.0)	**0.011**	58 (35.8)	0.410
- CC	219 (27.7)		256 (32.4)	
rs20541:				
- AA/GA	104 (32.6)	0.153	114 (35.9)	0.189
- GG	177 (27.9)		200 (31.6)	
Birth order				
- First	98 (27.7)	0.659	116 (32.8)	0.683
- Second	91 (30.0)		105 (34.8)	
- Third or higher	64 (31.1)		64 (31.1)	
- Latent	33 (47.8)		31 (44.9)	
Family Social Status Cluster*				
- High	22 (27.5)	0.328	22 (27.5)	0.363
- Middle	213 (28.9)		251 (34.1)	
- Low	45 (35.2)		38 (29.7)	
Environmental Tobacco Exposure**				
- ETS-0	142 (31.5)	0.463	160 (35.6)	0.237
- ETS-1	85 (28.3)		89 (29.7)	
- ETS-2	54 (27.1)		64 (32.2)	
Gender				
- Male	146 (30.5)	0.523	178 (37.2)	**0.006**
- Female	135 (28.5)		136 (28.7)	
Prematurity (weeks)				
- < 37	14 (53.9)	**0.008**	14 (53.9)	**0.032**
- ≥ 37	257 (28.4)		290 (32.1)	
Low Birth Weight (g)				
- < 2500	8 (24.2)	0.566	11 (33.3)	1.000
- ≥ 2500	265 (29.6)		291 (32.6)	
Maternal History of Atopy				
- Yes	100 (30.7)	0.600	125 (38.3)	**0.013**
- No	181 (28.9)		189 (30.2)	
Maternal IgE				
- > 100	65 (37.4)	0.009	73 (42.0)	**0.002**
- ≤ 100	130 (26.7)		140 (28.8)	
Maternal Atopic Status***				
- Definite	32 (30.5)	0.006	42 (40.0)	**0.014**
- Latent	33 (47.8)		31 (45.0)	
- Probable	31 (26.3)		35 (30.0)	
- None	99 (26.8)		105 (28.5)	
Cord Serum IgE				
- ≥ 0.5	34 (43.6)	0.004	37 (47.4)	**0.005**
- < 0.5	202 (27.4)		229 (31.0)	

*"Family social status cluster" is a composite variable derived from a combination of family income, parental occupation (socioeconomic status), and number of children in child's bedroom.

** ETS-0: mother did not smoke during pregnancy and children not exposed to household ETS; ETS-1: mother did not smoke during pregnancy, but children were exposed to household ETS; ETS-2: mother smoked during pregnancy and children were exposed to household ETS.

***Maternal atopic status was classified into Definite (elevated IgE, positive history); Latent (elevated IgE, negative history); Probable (normal IgE, positive history); and None (normal IgE, negative history).

**Table 4:** Univariate analysis of the association of positive skin prick test (SPT) with *IL13 *and prenatal factors

	Positive SPT (age 4)		Positive SPT (age 10)		Positive SPT (age 18)	
	n (%)	Exact	n (%)	Exact	n (%)	Exact
Study Variables	N = 981	p-values	N = 1,036	p-values	N = 845	p-values
rs1800925:						
- TT/CT	56 (21.8)	0.317	94 (28.7)	0.365	105 (43.6)	0.354
- CC	137 (18.9)		183 (25.9)		242 (40.1)	
rs2066960:						
- AA/AC	31 (23.9)	0.195	50 (30.7)	0.247	47 (39.8)	0.840
- CC	162 (19.0)		227 (26.0)		300 (41.3)	
rs20541:						
- AA/GA	54 (20.9)	0.584	89 (27.6)	0.705	98 (42.1)	0.754
- GG	139 (19.2)		188 (26.3)		249 (40.7)	
Birth order						
- First	84 (21.1)	0.549	107 (27.2)	0.950	130 (39.5)	0.121
- Second	67 (19.6)		89 (27.6)		116 (47.4)	
- Third or higher	42 (17.5)		57 (26.4)		73 (39.3)	
Family Social Status Cluster*						
- High	20 (23.8)	0.492	19 (22.9)	0.516	25 (36.8)	**0.023**
- Middle	150 (19.7)		221 (27.7)		276 (43.1)	
- Low	23 (17.2)		35 (24.3)		33 (29.7)	
Environmental Tobacco Exposure**						
- ETS-0	100 (21.9)	0.145	141 (28.9)	0.213	183 (43.8)	0.245
- ETS-1	63 (19.4)		86 (26.2)		97 (38.5)	
- ETS-2	30 (15.2)		49 (22.6)		63 (37.5)	
Gender						
- Male	109 (22.1)	0.055	158 (30.6)	**0.005**	188 (46.2)	**0.002**
- Female	84 (17.2)		119 (22.9)		156 (35.7)	
Prematurity (weeks)						
- < 37	5 (17.2)	1.000	12 (41.4)	0.086	9 (39.1)	1.000
- ≥ 37	183 (19.7)		257 (26.2)		321 (40.4)	
Low Birth Weight (g)						
- < 2500	4 (11.1)	0.283	11 (31.4)	0.559	9 (34.6)	0.685
- ≥ 2500	184 (20.0)		256 (26.3)		321 (40.7)	
Maternal History of Atopy						
- Yes	75 (22.1)	0.178	117 (32.7)	0.002	137 (47.6)	**0.005**
- No	118 (18.4)		160 (23.6)		207 (37.4)	
Maternal IgE						
- > 100	40 (22.4)	0.223	68 (34.3)	0.002	78 (48.8)	**0.002**
- ≤ 100	90 (18.0)		116 (22.4)		146 (34.5)	
Maternal Atopic Status***						
- Definite	20 (19.2)	0.355	40 (33.9)	0.010	48 (49.5)	**0.008**
- Latent	20 (26.7)		28 (35.0)		30 (47.6)	
- Probable	24 (18.8)		32 (25.0)		42 (40.0)	
- None	66 (17.7)		84 (21.5)		104 (32.7)	
Cord Serum IgE						
- ≥ 0.5	29 (32.2)	0.002	32 (35.6)	0.042	37 (50.0)	0.060
- < 0.5	134 (17.8)		201 (25.2)		253 (38.4)	

*"Family social status cluster" is a composite variable derived from a combination of family income, parental occupation (socioeconomic status), and number of children in child's bedroom.

** ETS-0: mother did not smoke during pregnancy and children not exposed to household ETS; ETS-1: mother did not smoke during pregnancy, but children were exposed to household ETS; ETS-2: mother smoked during pregnancy and children were exposed to household ETS.

***Maternal atopic status was classified into Definite (elevated IgE, positive history); Latent (elevated IgE, negative history); Probable (normal IgE, positive history); and None (normal IgE, negative history).

#### Multivariable analysis

##### Elevated serum IgE at age 10 years

Using multivariable log-linear regression analysis and after mutually adjusting for potential confounders, there was a significant interaction between rs1800925 and birth order on elevated serum total IgE at age 10 years (p = 0.023). To unfold this interaction, we stratified the analysis by birth order (Table 5). After stratification, the explanatory model showed the effect of rs1800925 on elevated serum total IgE at age 10 to be restricted only to first-born children (p = 0.007; adjusted Prevalence Ratio (PR) = 1.73; 95% CI = 1.16, 2.57). No other significant associations were found in this model (Table 5).

##### Serum specific inhalant IgE screen positivity at age 10 years

In the multivariable log-linear regression analysis and after mutually adjusting for potential confounding variables, we identified a statistically significant interaction between rs20541 and birth order on serum specific inhalant IgE positivity at age 10 (p = 0.029). To demonstrate this interaction, we stratified the analysis by birth order (Table 6). The stratified analysis showed the effect of *IL13 *on positive inhalant IgE screen at age 10 to be restricted only to first-born children (p = 0.034; adjusted PR = 1.48; 95% CI = 1.03, 2.13). No other significant associations were found.

**Table 5:** Multivariate analysis of the association of elevated total serum IgE at age 10 (>200 kU/L) with *IL13 *polymorphisms and prenatal factors, stratified by birth order (n = 588) (PR = Prevalence Ratio)

	1^st ^Born		2^nd ^Born		3^rd ^and Higher	
Study Variables	p	PR (95% CI)	p	PR (95% CI)	p	PR (95% CI)
	n = 246		n = 212		n = 130	
rs1800925:						
-TT/CT vs. CC	**0.007**	1.73 (1.16, 2.57)	0.165	0.72 (0.45, 1.15)	0.794	0.93 (0.54, 1.61)
						
Gender (M vs. F)	0.499	1.14 (0.77, 1.71)	0.208	1.31 (0.86, 1.99)	0.350	1.28 (0.76, 2.15)
						
Smoke exposure*:						
- ETS-1 vs. ETS-0	0.38	0.82 (0.53, 1.27)	0.175	0.70 (0.42, 1.17)	0.863	0.95 (0.54, 1.69)
- ETS-2 vs. ETS-0	0.064	0.54 (0.28, 1.04)	0.202	0.65 (0.33, 1.26)	0.536	0.79 (0.38, 1.66)
						
Family SES Cluster**:						
- Low vs. High	0.198	1.88 (0.72, 4.91)	0.840	1.09 (0.49, 2.40)	0.589	0.72 (0.21, 2.41)
- Middle vs. High	0.767	1.13 (0.50, 2.54)	0.379	0.76 (0.41, 1.41)	0.915	1.05 (0.43, 2.54)
						
Gestational Age	0.173	0.93 (0.83, 1.03)	0.246	1.09 (0.94, 1.28)	0.582	1.06 (0.86, 1.31)

* ETS-0: mother did not smoke during pregnancy and children not exposed to household ETS;

ETS-1: mother did not smoke during pregnancy, but children were exposed to household ETS;

ETS-2: mother smoked during pregnancy and children were exposed to household ETS.

**"Family social status cluster" is a composite variable derived from a combination of family income, parental occupation (socioeconomic status), and number of children in child's bedroom.

**Table 6:** Multivariate analysis of the association of inhalant serum IgE at age 10 with *IL13 *polymorphisms and prenatal factors, overall population, stratified by birth order (n = 588) (PR = Prevalence Ratio)

	1^st ^Born		2^nd ^Born		3^rd ^and Higher	
Study Variables	p	PR (95% CI)	p	PR (95% CI)	p	PR (95% CI)
	n = 246		n = 212		n = 130	
rs20541:						
-AA/GA vs. GG	**0.034**	1.48 (1.03, 2.13)	0.120	0.72 (0.48, 1.09)	0.197	1.43 (0.83, 2.45)
						
Gender (M vs. F)	0.353	1.19 (0.82, 1.72)	**0.017**	1.58 (1.08, 2.29)	0.496	1.20 (0.71, 2.02)
						
Smoke Exposure*:						
- ETS-1 vs. ETS-0	0.075	0.68 (0.44, 1.04)	0.154	0.73 (0.47, 1.13)	0.600	0.84 (0.45, 1.60)
- ETS-2 vs. ETS-0	0.318	0.79 (0.49, 1.26)	0.278	0.74 (0.43, 1.27)	0.672	1.14 (0.60, 2.19)
						
Family SES Cluster**						
- Low vs. High	0.070	2.27 (0.94, 5.49)	0.860	0.92 (0.35, 2.43)	0.058	0.23 (0.05, 1.05)
- Middle vs. High	0.435	1.37 (0.62, 3.04)	0.506	1.26 (0.64, 2.46)	0.349	0.69 (0.31, 1.51)
						
Gestational age	0.240	0.94 (0.85, 1.04)	0.649	1.03 (0.90, 1.18)	0.095	1.26 (0.96, 1.65)

* ETS-0: mother did not smoke during pregnancy and children not exposed to household ETS;

ETS-1: mother did not smoke during pregnancy, but children were exposed to household ETS;

ETS-2: mother smoked during pregnancy and children were exposed to household ETS.

**"Family social status cluster" is a composite variable derived from a combination of family income, parental occupation (socioeconomic status), and number of children in child's bedroom.

##### Skin prick tests at 4, 10 and 18 years: Cross-sectional analyses

We assessed the interaction of *IL13 *polymorphisms with SPT at the different time-points. Multivariable analysis showed significant interaction on a multiplicative scale between *IL13 *and birth order on SPT positivity at age 10 (rs20541, p = 0.07) and age 18 (rs20541, p = 0.030 and rs2066960, p = 0.027) but not at age 4. To further demonstrate and compare these effects on an additive scale, we stratified by birth order at each time-point. Among first-born children, minor allele carrier-ship of rs20541 showed a trend towards a higher risk of skin sensitization at age 4 (PR = 1.59; 95% CI = 1.01, 2.50), age 10 (PR = 1.48; 95% CI = 0.99, 2.20) and 18 years (PR = 1.21; 95% CI = 0.86, 1.69). On the other hand, there was no such increased risk of atopy among second-born children at age 4 (PR = 0.81; 95% CI = 0.43, 1.50), age 10 (PR = 0.70; 95% CI = 0.42, 1.15) and 18 years (PR = 0.76; 95% CI = 0.51, 1.14). Compared to the referent group, in children with a birth order of 3 or greater, there was also a higher but non-significant prevalence ratio of SPT in children with minor allele carriership of rs20541.

##### Skin prick tests at 4, 10 and 18 years: Repeatedmeasurement analysis

After confounder adjustment, the multivariable repeated measurement regression analysis showed a significant interaction between rs20541 and birth order on SPT at ages 4, 10 and 18 years at an alpha level of 0.1 (p = 0.076). To further describe this interaction, we stratified the statistical model by birth order (Table 7). Table 7 focuses on the effect of *IL13 *polymorphism on SPT within each birth order category. The stratified analysis showed that the effect of *IL13 *on SPT at ages 4, 10 and 18 was evident only among first-born children (p = 0.007; adjusted PR = 1.35; 95% CI = 1.09, 1.69), with borderline significance for second-born children. Children with a birth order of 3 and higher showed an increased but non-significant prevalence ratio of SPT positivity.

Comparing children from low socio-economic backgrounds with those from the high end of the social status scale, it is also evident from Tables 5 to 7 that there is a decreasing (though not statistically significant) trend of risk for atopy as birth order increases from one to three and above. First born children from low social status backgrounds had increased risk of atopy (PR = 1.51; 95% CI = 0.93, 2.45, not significant; Table 7), while third and higher order birth children were protected from the risk of atopy (PR = 0.34; 95% CI = 0.16, 0.73, p = 0.006; Table 7). In addition, gestational age was a significant predictor of skin test sensitivity for second and higher order birth children (p = 0.03 and 0.01 respectively; Table 7).

**Table 7:** Multivariate repeated measurement analysis of the association of positive skin prick test (SPT) at ages 4, 10 and 18 with *IL13 *and prenatal factors, stratified by birth order (n = 825; obs = 1,305) (PR = Prevalence Ratio)

	1^st ^Born		2^nd ^Born		3^rd ^and Higher	
Study Variables	p	PR (95% CI)	p	PR (95% CI)	p	PR (95% CI)
	(n = 354; obs = 772)^#^		(n = 287; obs = 634)		(n = 184; obs = 414)	
rs20541:						
-AA/GA vs. GG	**0.007**	1.35 (1.09, 1.69)	0.045	0.75 (0.56, 0.99)	0.075	1.35 (0.97, 1.89)
						
Gender (M vs. F)	**0.014**	1.32 (1.06, 1.66)	**0.001**	1.50 (1.18, 1.89)	0.082	1.34 (0.96, 1.85)
						
Environmental Smoke Exposure*:						
- ETS-1 vs. ETS-0	0.577	0.93 (0.73, 1.19)	0.698	1.05 (0.81, 1.36)	0.179	0.76 (0.52, 1.13)
- ETS-2 vs. ETS-0	**0.020**	0.65 (0.45, 0.93)	0.101	0.75 (0.53, 1.06)	0.309	0.79 (0.50, 1.25)
						
Family SES Cluster**:						
- Low vs. High	0.099	1.51 (0.93, 2.45)	0.331	0.73 (0.39, 1.38)	**0.006**	0.34 (0.16, 1.37)
- Middle vs. High	0.741	1.07 (0.70, 1.64)	0.355	1.24 (0.78, 1.97)	0.091	0.67 (0.43, 1.06)
						
Gestational Age	0.377	0.96 (0.89, 1.04)	**0.033**	1.10 (1.01, 1.21)	**0.010**	1.24 (1.05, 1.47)

^# ^354 children provided 772 observations because SPT was assessed at 3 separate time-points - 4, 10 and 18 years.

* ETS-0: mother did not smoke during pregnancy and children not exposed to household ETS;

ETS-1: mother did not smoke during pregnancy, but children were exposed to household ETS;

ETS-2: mother smoked during pregnancy and children were exposed to household ETS.

**"Family social status cluster" is a composite variable derived from a combination of family income, parental occupation (socioeconomic status), and number of children in child's bedroom.

## Discussion

Our analyses showed a statistically significant interaction between *IL13 *polymorphisms and birth order for elevated serum IgE at age 10, serum inhalant specific IgE positivity at age 10, and for SPT at ages 4, 10 and 18 years. An interaction on an additive scale was found both in the cross-sectional analysis and in the repeated measurement analysis. The predictive value of *IL13 *genotypes on the atopic markers was restricted only to first born children.

Our findings do not result from a selection bias for several reasons. First, the study cohort maintained high follow-up proportions throughout the entire study period: 83.7% (1,218/1456) at age 4; 94.3% (1,373/1456) at age 10; and 89.6% (1,304/1456) at age 18 years. Secondly, children who had IgE measurement results differed only with regard to environmental tobacco smoke exposure and family social status cluster from those who had no IgE measurements (Table 2). With respect to the genotypes, the presence of a selection bias could result in a violation of the Hardy-Weinberg law. The genotypes of the five *IL13 *SNPs were in Hardy-Weinberg equilibrium and their allele frequencies were comparable with those of other Caucasian populations [37,39,40] (Table 1). Hence, concerning the genetic polymorphisms, a selection bias is unlikely. In addition, due to the commencement of recruitment and assessments pre-natally, and the use of both questionnaires and physical examinations for obtaining information from the participants and their parents, information bias, if present, was minimal and non-differential. Non-differential information bias implies that any bias present would be similar in both affected and unaffected children, leading to a bias of the effect estimates towards the null.

While associations with different SNPs on the same gene are not considered an indication of disagreement [1,41,42], we found that two SNPs were related to atopic outcomes assessed in this study. Thus, our finding that different SNPs were associated with elevated total serum IgE (rs1800925), and with inhalant IgE and SPT (rs20541) is not surprising. Indeed, it is possible that total serum IgE is an indicator of *general *susceptibility (rs1800925 is at the promoter region of *IL13*), while the more *specific *reactions to inhalant allergens and skin sensitization are related to the non-synonymous (functional) SNP, rs20541 (exonic SNP).

Birth order [10,14,15] and *IL13 *polymorphisms [16-21] have each been previously found to be separately associated with allergy, asthma and atopic markers in childhood. However, this is the first study that shows an *interaction *between birth order and *IL13 *polymorphisms. If confirmed by other researchers, this effect modification may in part explain the mechanism of the birth order effect: genetic polymorphisms in the *IL13 *gene may undergo epigenetic changes *in utero *due to conditions specific to a first pregnancy compared to subsequent pregnancies. It is now established that the DNA provides the blueprint for the manufacture of all the proteins necessary to create a living organism. Nonetheless, epigenetic modifications provide additional instructions on how, where, and when the genetic information will be used (gene expression). These epigenetic changes generally involve DNA modification such as methylation and acetylation, histone protein modifications, and regulation of gene expression by microRNAs [43]. Such regulatory mechanisms specify which regions of the genome are active in any given cell at any one point in time [44,45]. Inherited changes in the "epigenome" have been postulated as a possible pathway explaining the differences in gene expression seen in individuals with identical "genomes" [46]. Such *in utero *epigenetic modifications may result in a higher susceptibility to increased total IgE levels and allergic sensitization in first-born children compared to children of higher-order births.

We have shown that the effect of *IL13 *polymorphism was restricted to firstborn children, with individuals carrying the homozygous minor and heterozygous genotypes having a higher relative risk (prevalence ratio) *among firstborns*. In addition, there was a lowered relative risk of the rs20451 SNP in second born, and a higher relative risk in children with a birth order of three or greater, although these were not statistically significant. The recurrence of an increased relative risk in children with 3^rd ^or higher order births was not expected. However, since this group is comprised of children with different birth orders (three and higher), it is possible that a long inter-pregnancy interval may modify the intrauterine environment. In support of this hypothesis, Wegienka et al. [47] found that children born after inter-pregnancy intervals of less than 2 years were less likely to have a positive SPT result compared with children of mothers with no prior pregnancies. Since a successful pregnancy is a fine balance between a mother's tolerance for her fetus' foreign genetic material and the fetus' ability to survive the maternal immunologic defenses, Wegienka et al. suggested that prior pregnancies and their spacing might influence the intrauterine environment. It is possible that closely spaced pregnancies may provide a higher maternal tolerance.

Our findings suggest adverse prenatal programming in firstborn offspring. This impact may be related to immunological differences between a first-born pregnancy and later pregnancies from the same partner, as has been previously documented for the etiology of preeclampsia [13]. However, in order to distinguish the concept of hygiene hypothesis from that of prenatal programming, the greater prevalence of atopy in firstborns may warrant further investigation. To clarify this issue, one needs to investigate whether maternal IgE or cord serum IgE modifies the interaction between birth order and allergic sensitization. If these prenatal markers are shown to be effect modifiers, then the observed effect is more likely to be associated with prenatal exposures than with post-natal infections.

The advantages of a birth cohort with prolonged follow-up, such as the Isle of Wight cohort, has been discussed in the literature as a necessary means to increase our understanding of genetic diseases, especially atopic disorders [48]. Unlike other studies that assessed cord blood IgE at birth as the outcome [49] or that measured child IgE after a few months or years of follow up [50], children in the Isle of Wight birth cohort were recruited prenatally and followed to age 18 years, including repeated assessments at ages 4, 10 and 18 years. Since allergy and atopy are dynamic manifestations that can vary with age due to recoveries and relapses, the long-term predictive value of the majority of previous studies was hampered by the short-term follow up period. The use of data up to age 18 years in this paper addresses this gap.

Finally, future functional genetic research, including gene expression and methylation studies, need to determine whether birth order is related to altered expression or methylation of the *IL13 *gene in relation to atopic outcomes. If we know that the methylation status is affected, it may be possible to develop interventions *during pregnancy *that could change global or *IL13*-specific methylation. Thus, these findings may inform research into allergy prevention by identifying which specific conditions may modify the altered expression of the *IL13 *gene in firstborns when compared to second borns.

## Competing interests

The authors declare that they have no competing interests.

## Authors' contributions

IUO conceived of the manuscript idea, drafted the manuscript and performed the statistical analysis. WJK provided supervision and guidance in the design, analysis and writing of the manuscript. HZ provided statistical oversight while TSA assisted with the interpretation of genetic results. SE, GR and SHA participated in the design and coordination of the original cohort study, carried out the molecular genetic studies, provided oversight in interpretation of analytic results and participated in all stages of the manuscript writing process. All authors read and approved the final manuscript.
